# Long-range synchrony and emergence of neural reentry

**DOI:** 10.1038/srep36837

**Published:** 2016-11-22

**Authors:** Hanna Keren, Shimon Marom

**Affiliations:** 1 Network Biology Research Laboratory, Electrical Engineering, Technion- Israel Institute of Technology, Haifa 3200003, Israel; 2 Department of Physiology, Biophysics and Systems Biology, Technion - Israel Institute of Technology, Haifa 32000, Israel

## Abstract

Neural synchronization across long distances is a functionally important phenomenon in health and disease. In order to access the basis of different modes of long-range synchrony, we monitor spiking activities over centimetre scale in cortical networks and show that the mode of synchrony depends upon a length scale, λ, which is the minimal path that activity should propagate through to find its point of origin ready for reactivation. When λ is larger than the physical dimension of the network, distant neuronal populations operate synchronously, giving rise to irregularly occurring network-wide events that last hundreds of milliseconds to several seconds. In contrast, when λ approaches the dimension of the network, a continuous self-sustained reentry propagation emerges, a regular seizure-like mode that is marked by precise spatiotemporal patterns (‘synfire chains’) and may last many minutes. Termination of a reentry phase is preceded by a decrease of propagation speed to a halt. Stimulation decreases both propagation speed and λ values, which modifies the synchrony mode respectively. The results contribute to the understanding of the origin and termination of different modes of neural synchrony as well as their long-range spatial patterns, while hopefully catering to manipulation of the phenomena in pathological conditions.

Spontaneous synchronization between remote neural networks is one of the hallmarks of brain activity, considered significant for multiple functions in health and disease^1^,^2^,^3^,^4^,^5^,^6^. As such, the study of mechanisms underlying long-range synchronized activity is of substantial interest. In the cortex, centimetre scale synchronization involves an interplay between diverse excitatory and inhibitory connectivities, ranging from broadly distributed statistics of synaptic connectivity amongst excitatory neurons to the global effect of rapidly propagated activity through electrically coupled inhibitory neurons^7^,^8^,^9^,^10^,^11^.

There are many different *microscopic parameters* at the cellular and synaptic levels that impact on the above interplay between excitation and inhibition^12^,^13^,^14^,^15^. But here we are interested in exposing a *global physical parameter* that mediates the translation of the many microscopic mechanisms to macroscopic modes of long-range synchrony. Such parametrisation has been useful in analysing cardiac reentry arrhythmias^16^; measurements of cyclic brain activity suggest that a similar approach might be instructive for the case of neural synchrony^17^.

To this aim, we take advantage of a reduced experimental model of large-scale networks composed of randomly connected cortical neurons. We show that the mode of synchrony is sensitive to a characteristic length scale, *λ*, which is the product of two parameters – the time scale (*τ*) of the individual synchronous event (including its refractory period), multiplied by the speed of activity propagation (*v*). When this length scale exceeds the longest propagation path offered by the network, the system acts as a single compartment, being active simultaneously. In contrast, when the characteristic length scale is in the range of propagation paths supported by the network, self-sustained activities appear, similar to the familiar reentrant dynamics in cardiac arrhythmia.

We use a pharmacological blockade of GABA_*A*_ mediated inhibition, well documented as inducing abnormal seizure-like synchronization^18^,^19^,^20^, and show that the relevant parameters (*τ* and *v*) are manipulated and their relations determine the resulting global mode of synchrony: from network-wide simultaneous activity that occurs irregularly, to regular ongoing reentry dynamics that may last many minutes, while running through temporally precise synfire-chains. Moreover we analyze the condition for termination of a reentry phase and show the impact of stimulation on these features.

## Results and Discussion

### Long-range synchronization

**Figure 1:**
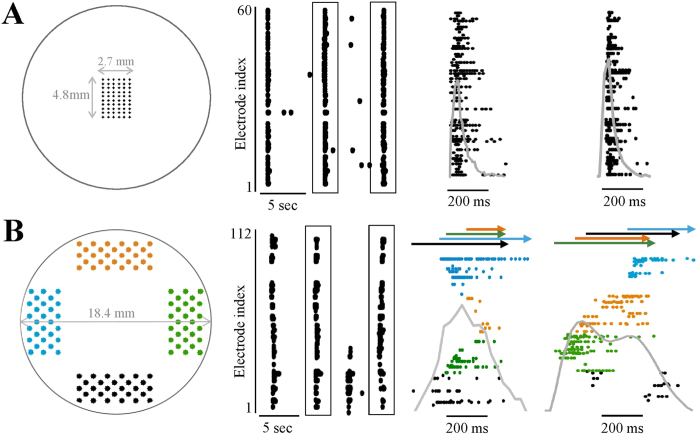
Recording long-range synchronization. (**A**) Scheme of a standard centred array of 60 electrodes. Three examples of spiking activity monitored through such an array are shown at the middle panel. Each point depicts a single spike detected in one of the electrodes (indexed in the vertical axis). Two synchronous events (boxed) are presented at higher temporal resolution on the right-hand panel. A superposed grey line shows normalized summed activity (binned to 25 ms). (**B**) Similar presentation of activity recorded using a layout of 112 electrodes arranged in four clusters of recording regions (color coded). Arrows demonstrate the duration of activation in each of the regions in respective colours.

When allowed to develop for a couple of weeks outside the brain, a population of cortical neurons (extracted from the rat newborn) tends to form a large-scale network that exhibits complex spontaneous activity. This spontaneous activity is characteristically composed of synchronous events (a.k.a. ‘network spikes’) that occur irregularly at an average rate of ca. 0.1 Hz, interspersed with some uncorrelated sporadic activities^21^,^22^,^23^,^24^,^25^,^26^. The temporal envelope of each synchronous event is usually monitored by integrating the spiking activities detected through many individual electrodes (in the order of several tens) arranged in an array. In most cases the millimetre scale electrode array is positioned at the centre of the large-scale network (Fig. 1A, left panel). Monitored through such relatively dense array of electrodes at the centre, the time scale of a single synchronous event – a single network spike – is in the order of one hundred milliseconds, as depicted in Fig. 1A (middle and right panels) and discussed elsewhere^25^.

In order to adjust the above standard experimental design to the subject matter of the present study that involves distances an order of magnitude larger, we constructed an electrode array layout consisting of four clusters that cover a significantly wider area (Fig. 1B, left panel; different colours depict the physical identity of each recording region). As can be appreciated by the examples provided in the middle and right panels of Fig. 1B, when activity is integrated over such distances, the apparent time scale of a single synchronous event is accordingly extended (1.02 ± 0.47 seconds; n = 1721, 8 networks), a value that is comparable to durations observed *in-vivo*^9^,^27^.

**Figure 2:**
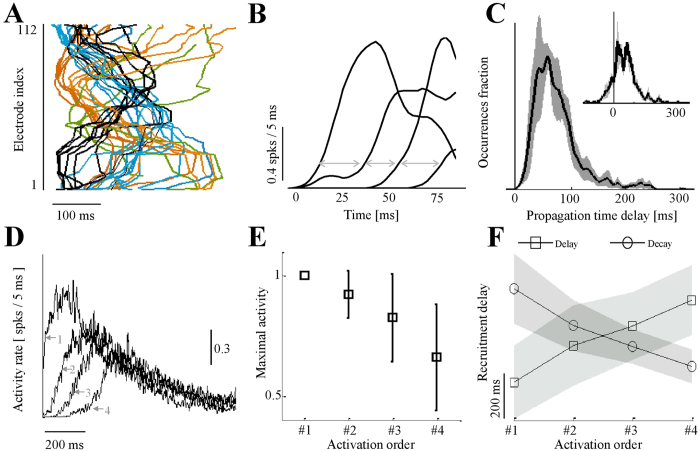
Propagation and termination of synchronized activity across remote regions. (**A**) Several synchronous events (32) presented according to the temporal order (horizontal axis) of the participating electrodes (vertical axis). Color is indicative of the identity of the recording region where synchrony was initiated. (**B**) Propagation delays between recording regions, exemplified for a single synchronous event, presented as cumulative activity of each of the recording regions (grey arrows). The distribution of such delays between initiation times of temporally adjacent regions of all synchronous events (see Methods), is shown in panel C (average of distributions of eight networks, 1721 events; standard deviation across networks depicted by shaded area). Inset: distribution of delays calculated between times of maximal activity of regions, but sorted according to the respective initiation time of each region. Note negative values that represent a mismatch between the order of initiation and the order of arriving at maximal activity. (**D**) All synchronous events (n = 1721, 8 networks), averaged and classified according to the rank-order of initiation times. (**E**) Values of maximal activity for each region (calculated per synchrony as the maximal count of spikes in 25 ms time bins), averaged and ranked according to initiation times. For each event, values of second (#2) to fourth (#4) recruited regions are normalized to maximal activity of the first-to-fire region (#1). Error bars represent standard deviation across all events. (**F**) Time delay from detection of initiation of activity in a given recording region, to maximal activity in that same region; this value (square symbols) is a proxy for within-region recruitment rate. Circles depict time from maximal activity of a given region to first occurrence of no activity within that same region, indicating within-region decay time. Shaded margins are standard deviation across all events.

The region where from synchronous activity is initiated varies between synchronous events. Figure 2A shows that the path of propagation amongst the four regions is uniquely determined by the region of activity initiation; this is indicated by the recruitment profiles of 32 synchronous events of a single network, color coded according to initiation region. The delay between activities recorded in different regions is exemplified for a single synchronization in Fig. 2B (calculated by comparing the initiation time of activity in each of the recording regions as described in the Methods). The values of all such delays across the eight different networks studied here, are consistent, as shown by the average distribution across all networks in Fig. 2C (1721 events); the average delay is found to be 80 milliseconds. The calculated speed of propagation (*v*) is 0.18 meter per second (SD = 0.08, n = 1721 synchronous events), which is within the range of reported propagation speed in the primate cortex^28^. The interval (*τ*) between two subsequent initiations of network spikes, composed of the synchronous event itself and the recovery period that follows, is broadly distributed, spanning a range of several seconds (*τ* = 10.3 seconds, SD = 11, n = 1721). The standard deviation of *τ*, which is in the order of the mean, is mainly contributed by variations in the inter-event-intervals rather than the actual network spike shape. The minimal value of *τ* is rarely (<5%) shorter than 3–4 seconds^25^,^26^,^29^.

Note that the complex nature of connectivity statistics may be appreciated by observing that earlier spiking activity in a given region does not entail a respective earlier peak of the synchronous event within that region. In 17% of the 1721 network spikes examined, a synchronous event initiated in one region reaches peak population activity in a downstream region earlier than in its upstream originator (indicated by negative values in the inset to Fig. 2C, where the distribution is of delays between times of maximal activity of regions, sorted according to the initiation time of each region). This observation might reflect a complex graph of connectivity and the presence of hub neurons; as it has been reported previously^25^,^30^,^31^,^32^,^33^,^34^,^35^, as well using using the same experimental setup, a complex connectivity graph gives rise to such “paradoxical” time delays.

The product *τ*·*v* yields a characteristic length scale (*λ*) that sets a constraint on the possible mode of synchrony. It provides an estimate for the minimal length of path through which activity should travel before meeting its point of origin ready for reactivation. The networks studied here are randomly spread on top of a circular plate having a perimeter of ≈6 centimetres. Therefore, the numbers provided above (*τ* > 3 seconds, *v* ≈ 20 centimetre per second) yield a characteristic length that is more than an order of magnitude larger compared to the dimensions of the system, entailing a single-compartment-like behaviour.

Consistent with the above, the results presented in Fig. 2D–F demonstrate simultaneous termination of synchronous activity at distant sites on a centimetre scale. In Fig. 2D, four traces of synchronous events – averaged from 8 different networks – are shown, classified according to the *rank-order of activation times*: The leftmost trace (depicted by arrow 1) is the average activity (spikes per millisecond) recorded in the first-to-synchronize region, regardless of its physical location. Note that, congruent with the data of Fig. 2A, different regions contributed to the average of this leftmost trace, as each global synchrony event could have originated in any one of four different physical regions. Likewise, the traces depicted by arrows 2–4 are the average activities recorded in the second, third and fourth-to-synchronize regions, respectively, regardless of their physical identities. On *average*, the envelope of a global synchronous event is dictated by the first-to-synchronize region; synchronized activity in all downstream regions converge to that envelope. This is also manifested as decreased amplitudes and durations of synchronized events in downstream regions (Fig. 2E,F). Overall, in spite of consistent offset in the initiation of synchronized activity – fully accounted for by propagation delays – on average there is no appreciable offset in the termination of remote activities; the process of synchrony cessation over relatively long distances seems literally simultaneous.

The phenomenon of simultaneous termination, without which a large-scale network can fall into modes of self-sustained waves, has been hitherto demonstrated in several animals *in-vivo*, using single and multi-unit recordings, as well as EEG, and suggested to occur due to the inhibitory cells being strongly coupled via gap junctions^11^,^36^.

### Manipulating the characteristic length scale by disinhibition

**Figure 3:**
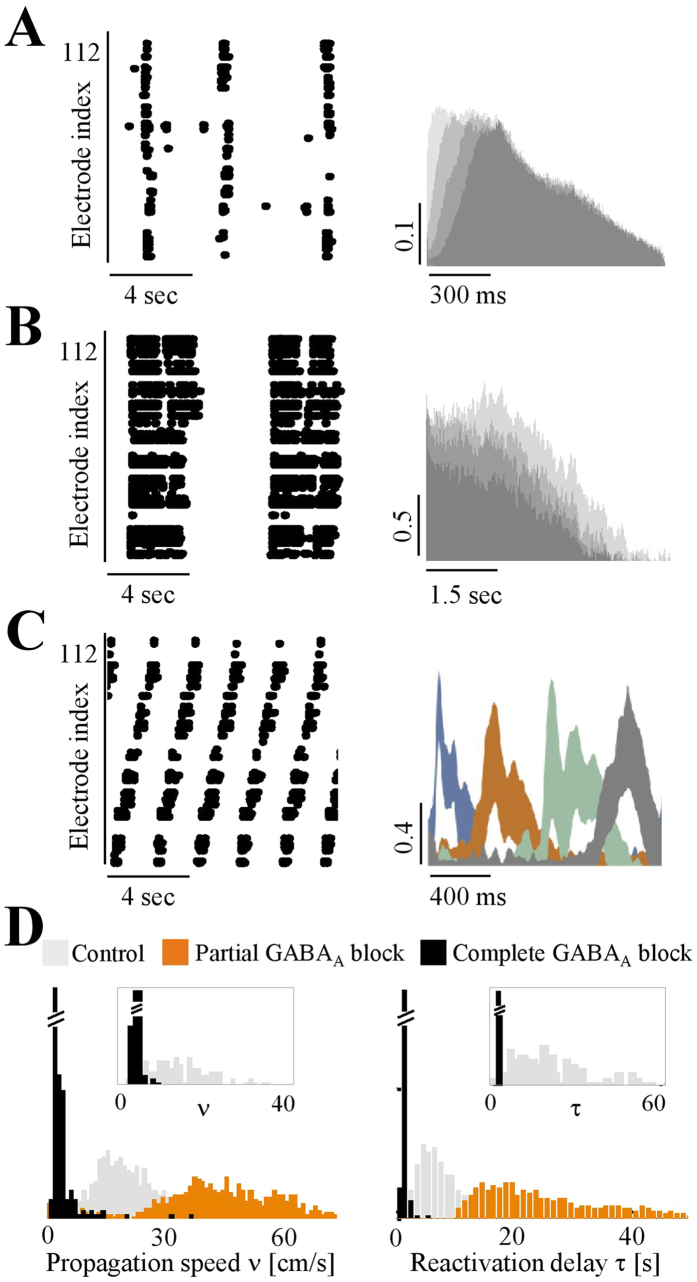
Manipulating characteristic length scale by disinhibition. (**A**) Left: Synchronous events under no disinhibition; each point depicts a single spike detected in one of the electrodes (indexed in the vertical axis). Right: All synchronous events (n = 1721, 8 networks), averaged and classified according to the rank-order of initiation times. For visual clarity only the positive margins of the standard deviation across all events are presented. (**B**) Synchronous events under *partial* Bicuculline block (5 *μ*M). Right: 146 events from a single network under this condition, classified and ranked according to region initiation time; only the positive margins of the standard deviation across all events are presented. (**C**) Example of activity under *complete* Bicuculline block. Right: standard deviation margins of 114 events under this condition. (**D**) Distributions of propagation speed *v* (left) and reactivation delays *τ* (right) under three conditions: *control* (no blockers; light grey, n = 1721, 8 networks), *partial GABA*_*A*_ *block* (5 *μ*M Bicuculline; orange, n = 1143, 8 networks) and *complete GABA*_*A*_ *block* (mean concentration of 100 *μ*M Bicuculline; black, n = 478, 8 networks). Propagation speed was estimated from the initiation delays between recording regions; histograms constructed with 1 cm/sec bins. Reactivation delays are the intervals between subsequent synchronous events across the whole network; histograms constructed with bins of 600 ms. Insets are the corresponding analyses of 3 networks with complete Picrotoxin block (200 *μ*M; black, n = 347) and control (light grey, n = 157).

We found GABA_*A*_ blockers (Bicuculline or Picrotoxin) very useful as means to explore the effects of the product *τ*·*v* on modes of synchrony. This is due to the fact of both blockers demonstrating a seemingly paradoxical effect of increasing *τ*·*v* at low concentrations, while decreasing it at high concentrations. As demonstrated in Fig. 3A,B, partial GABA_*A*_ blockade using ca. 5 *μ*M Bicuculline (IC50 = 3 *μ*M) significantly prolongs the duration of synchronized activity, from the range of hundreds of milliseconds to several seconds (3.69 ± 1.41 seconds; n = 1721, 8 networks). Recruitment toward synchrony becomes faster and propagation speed *v* is increased to 0.43 meter per second (SD = 0.19, n = 1143 events from 8 networks). The values of *τ* are also increased (average 28 seconds, SD = 16, n = 1143, 8 networks) and, as a consequence, the characteristic length scale becomes very large compared to control condition. The resulting network-wide synchrony mode is marked by extended (several seconds) relaxations. Termination across the network, while less simultaneous in absolute terms, is not much different from control condition when variation is normalized to the mean duration of the network spike. These observations are congruent with previous reports on the effect of GABA_*A*_ disinhibition in similar concentrations^25^,^36^.

Intriguingly, under different settings, other (even opposite) impacts of disinhibition on the duration of synchronization were reported^37^,^38^. Maybe related, Chen *et al*.^36^ suggested, based on numeric simulations, that further elimination of inhibition might give rise to an opposite effect of *reduced* duration of synchronous events, due to fast activation of hyperpolarizing conductances. With this in mind, we attempted pushing the product *τ*·*v* to values lower compared to control condition. Indeed, we observe a non-monotonous effect of Bicuculline as concentration of the blocker is further increased (Fig. 3C). Stronger GABA_*A*_ block by Bicuculline slows down the propagation speed (*v*) to 0.042 meter per second (SD = 0.015, n = 478, 8 networks); this is reflected in longer delays between activities of adjacent regions (Fig. 3D, left). In addition, at these higher concentrations, the blocker decreases the average *τ* to 1.52 seconds (SD = 0.44, n = 478, 8 networks); this is due to a counterintuitive shortening of within-region synchronous event duration, as well as shortening of the inter synchronous events intervals (Fig. 3D, right). We note a marked variability in the concentration of Bicuculline required to elicit these effects in the four networks tested here, the average being 100 *μ*M. The mechanism underlying the above non-monotonous effects of Bicuculine is not clear and published reports cast doubt on whether at all they are related to inhibitory synaptic transmission. Specifically, Bicuculline (methiodide derivative) has been shown to impact on membrane ionic conductances by blocking calcium-activated potassium channels^39^. We therefore replicated the main observations related to high Bicuculline concentration using Picrotoxin – a different class of GABA_*A*_ block^39^,^40^ – using a concentration indicated to induce complete blockade of GABA_*A*_ inhibition of 200 *μ*M^41^. Insets of Fig. 3D show that the effect of Picrotoxin on both propagation speed *v* and reactivation delays *τ* is similar to that of high concentrations of Bicuculline, suggestive of a possible mechanism that does involve inhibition. Regardless of the machinery underlying the effect of high Bicuculline or Picrotoxin concentrations, from the perspective of the present report what matters is that the synchronization mode under these conditions is characterized by slowly propagated activity that runs locally through adjacent regions (Fig. 3C).

### Reentry mode

**Figure 4:**
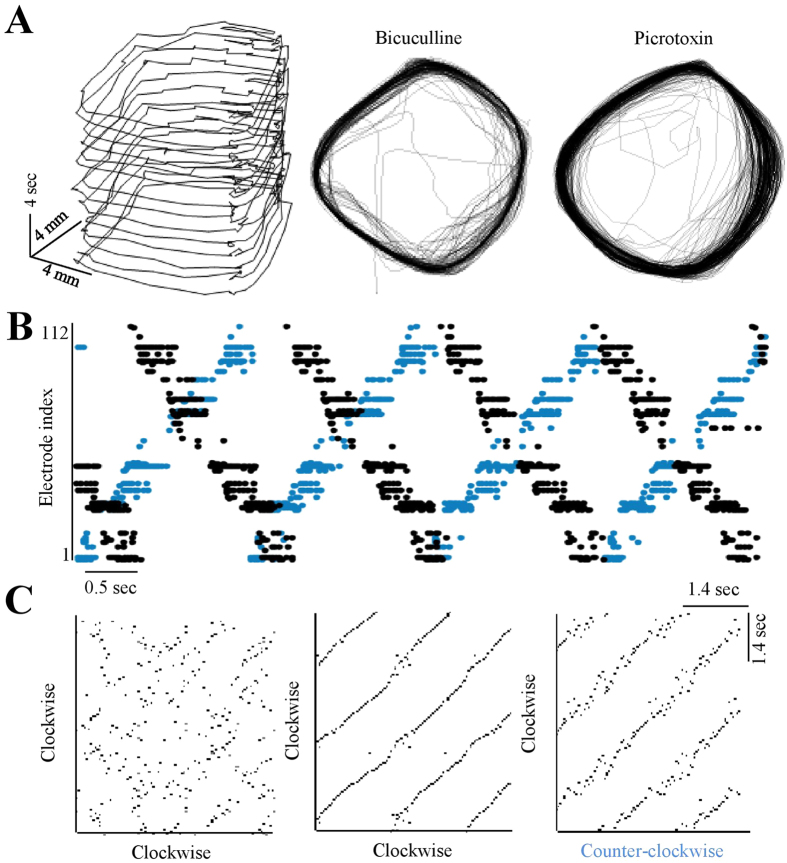
Reentry in disinhibited networks. (**A**) Coordinates of average activity position during reentry, calculated from the first spike detected by each electrode per cycle (moving average over 5 recent spikes). The right hand panels present two entire phases of reentry in other networks under complete Bicuculline or Picrotoxin block, lasting 3.3 and 8.3 min. (**B**) Two frames extracted from two consecutive phases of reentry in the same network; the phases run in opposite spatial directions (color coded). (**C**) Synfire chains detection procedure using a pair-wise similarity matrix following Schrader *et al*.^42^: Each pixel represents the level of similarity (scaled 0–1) between sets of active electrodes in two time bins (3 ms). Black depicts full identity (see Methods). The left-hand panel is calculated under control condition. The middle panel shows synfire chains detected in a clock-wise directed reentry activity. The right-side of panel of C is a comparison between a reversed *counter clock-wise* reentry propagation and a clock-wise data.

More specifically, complete GABA_*A*_ block reduces the product *τ*·*v* from ca. 200 cm in control conditions to lower values which at times are close to the physical perimeter of the network (slightly larger than 6 cm). Under these conditions, reentrant – self sustained (reverberating) patterns – emerge, and Fig. 4 (as well as videos S1, S2) demonstrates several realizations of such a mode. Phases of reentry may last many minutes (Fig. 4A), and the direction of the reentrant activity may flip between clockwise to counter-clockwise (Fig. 4B). The preciseness of the spatial and temporal patterns emerging under this condition is demonstrated in Fig. 4C using terms borrowed from Schrader *et al*.^42^ in their analyses aimed at identification of synfire-chains^43^,^44^. Intriguingly, comparison of activity patterns of clockwise and counter-clockwise reverberations (in a given network) suggests that waves of activity travel through (more-or-less) the same path, but in opposite directions (Fig. 4C right panel). This implies that under complete disinhibition, the spatial correlation length is shorter than the ca. 1 millimetre that separates nearby electrodes within a region.

**Figure 5:**
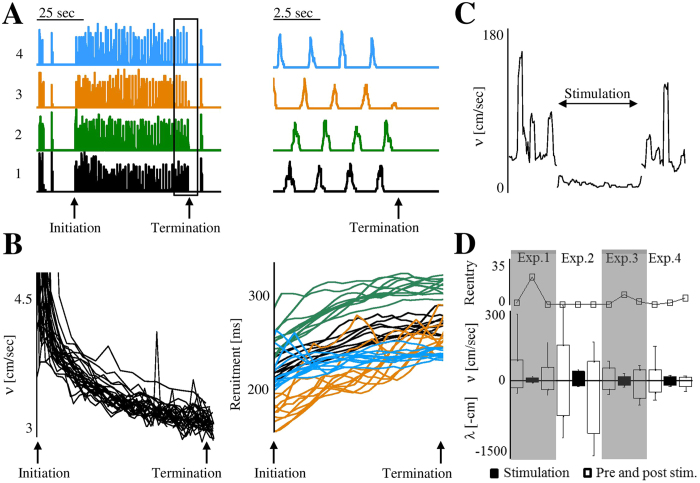
Termination of reentry phases. (**A**) The case of one reentry phase; the termination phase (boxed) is shown on the right panel at higher temporal resolution. (**B**) Left: Decline of propagation speed in 27 reentry phases of the same network, aligned according to initiation and termination times of each reentry phase. Right: Similar presentation of recruitment duration, estimated for each synchronous event from the time elapsed until all participating electrodes of a given region (color coded) detected at least one spike. (**C**) Propagation speed values in a single network during three recording phases: a pre-stimulation phase (with no reentry occurrences), stimulation (5 Hz, applied consecutively to all electrodes of a single recording region) and post-stimulation (horizontal axis is synchrony index). Each of the phases lasted for more than twenty minutes. (**D**) The same three recording phases in four experiments, for each the average values of *λ* (negative scale imposed for visual clarity), propagation speed and a count of reentry cycles are vertically presented.

Termination of a reentry phase occurs abruptly, as shown in Fig. 5A. As proposed by Golomb and Amitai^45^, network activity comes to a halt once propagation speed is below 5 centimetre per second, due to reduced excitatory synapses efficacy (of type AMPA). Indeed, in our case (Fig. 5B) termination is preceded by a gradual decrease of propagation speed to a threshold value of ca. 3 centimetre per second (mean = 3.42, SD = 0.26, across 44 reentry phases in 6 different networks), as well as a gradual decrease in population recruitment rate (Fig. 5B, right panel).

From an applicative context, this suggests that decreasing propagation speed below the threshold value could terminate a reentry phase. Stimulation, indicated as decreasing neural excitatory resources^46^, was shown to terminate abnormal epileptic activation when applied following the initiation of a seizure^47^. We find that applying frequent spread stimulation does cause an immediate decrease of propagation speed values (as exemplified in Fig. 5C,D). In parallel, stimulation induces a reduction of the length scale (*λ*) values (Fig. 5D), along a reduction in *τ* values and its variability (*τ* mean value during stimulation being 34% and 27% of the pre-stimulation and post-stimulation phases mean values, respectively; while the standard deviation of *τ* being 19% and 17%, respectively). These alterations result in the emergence of a more regular-frequent synchrony mode in all cases, converging to reentry once *λ* values are low enough (see Fig. 5D, two shaded cases; in these examples the lowest values of *λ* during stimulation are indeed in the range of network dimension: 6 cm and 7 cm, in contrary to the other two examples). Hence, stimulation can cause a state-dependent impact on the synchrony mode as applying stimulation in phases with no reentry (high propagation speed values), does not necessarily reach the low boundary of activation, but might induce reentry.

**Figure 6:**
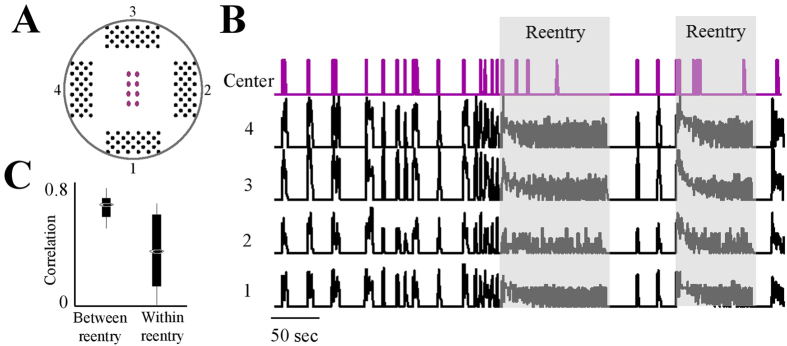
Activity detected in the central area during reentry propagation. (**A**) A scheme of a recording array that includes 8 electrodes in the central region. (**B**) Exemplar activities detected in the four peripheral regions (black) and one electrode located in the centre (purple), under complete Bicuculline block. Activity was binned to 250 ms. In between reentry phases, the activity detected in central electrodes is highly correlated with activity in peripheral electrodes; within reentry, this correlation drops. This is shown in panel (C) summarizing results from 11 central electrodes in 6 different networks.

And, finally, in a case where reentry waves run along the perimeter of a circular network, activity at the centre area is expected to be less stereotypic. Indeed, when reentry commences, the activity recorded from several electrodes located at the centre of the array is significantly reduced, and becomes less correlated with the activity picked by the peripheral electrodes (Fig. 6).

**Concluding remarks: (1)** By implementing a centimetre scale *in-vitro* experimental design and manipulating *GABA*_*A*_ mediated inhibitory activity, we demonstrate that a characteristic length scale, *λ* – the product of the time scale (*τ*) of a single synchronous event (including refractoriness) and the speed of activity propagation (*v*) – determines the mode of synchrony between distant network areas. When *λ* is in the range of the longest propagation path in a random network, dictated by the network dimensions, the network tends to fall into a self-sustained reentrant mode of synchronous activity. We further show that the length scale is sensitive, in a non-trivial manner, to the level of inhibitory transmission.

Disinhibition by blockade of *GABA*_*A*_ synaptic transmission is acknowledged to induce altered seizure-like synchronization, both *in-vivo* as well as *in-vitro*^9^,^18^,^19^,^20^,^36^. Among the described characteristics of such emerging epileptic-form synchronization, there has been some controversial observations, varying between regular short synchrony events - occurring in frequencies and temporal scales similar to the described reentry propagation - to occurrence of irregular activities^9^,^18^,^19^,^20^,^36^. Hence, this study also demonstrates that **(2)** Sampling at a relevant spatial scale matters, as interpretation of underlying mechanisms and spatiotemporal dynamics might be biased when partially monitored. For instance, when focusing on one peripheral region we might have interpreted reentry as phases of a focal source of short, regular synchrony events, rather than spatially spread continuous propagation. Likewise, we and others insisted, for many years now, to record the activity of large-scale random networks through a cluster of electrodes positioned in the centre of the network, thus avoiding boundary effects. Given the results reported here, focusing on activity at the centre of a disinhibited network would have led to an observation of irregular activities, masking the coherent nature of ongoing well-structured reentry dynamics, that can only be captured by spreading recording electrodes over the appropriate scale. Additionally, these results suggest a compilation of several observations of precisely timed propagation patterns^48^,^49^, to the realization of spatiotemporal features of reentry. **(3)** From an applicative context, the parametrization provided here enables the estimation of the spatial extent of such self-sustained reentry propagation, using generic activity parameters. Moreover, we show that termination of reentry is preceded by a decrease of propagation speed to a halt, also being reduced by applying stimulation. Epileptic activations have been shown to terminate once stimulation is applied following seizure’s initiation^47^. Here, the advantage of state-dependent stimulation protocols is exemplified from a length-scale perspective: stimulation impacts consistently on length scale parameters which results in a respective, non-monotonic impact on the synchrony mode, possibly also inducing reentry. And, **(4)** The results of our experimental analyses are not confined to the actual physical structure used here (circular arrangement); what matters is the ratio between the characteristic length scale and the longest propagation path supported by a network. Indeed, spontaneous occurrence of repeated cyclic propagation has been detected also *in-vivo*, in the absence of clear physical boundary conditions^17^. Therefore, the messages conveyed are relevant to discussions on the origin and termination of reverberating activity in normal and pathological conditions, described both *in-vivo* as well as *in-vitro*^6^,^38^,^50^,^51^,^52^,^53^,^54^,^55^,^56^,^57^,^58^,^59^.

## Methods

### Cell preparation

Cortical neurons were obtained from newborn rats (Sprague-Dawley) within 24 hours after birth using mechanical and enzymatic procedures described in earlier studies^23^. Rats were anesthetized by CO_2_ inhalation according to protocols approved by the Technion’s ethics committee. The neurons were plated directly onto substrate-integrated multi electrode arrays and allowed to develop into functionally and structurally mature networks over a period of 2–3 weeks. The number of plated neurons was of the order of 450,000, covering an area of 380 mm^2^. The preparations were bathed in MEM supplemented with heat-inactivated horse serum (5%), glutamine (0.5 mM), glucose (20 mM), and gentamycin (10 *μ*g/ml), and maintained in an atmosphere of 37 °C, 5% CO_2_ and 95% air in an incubator as well as during the recording phases. Inhibitory synaptic transmission was blocked by acute application to the bathing solution of either Bicuculline-Methiodide or Picrotoxin (Sigma-Aldrich).

### Electrophysiology

An array of 112 TiN extracellular electrodes of 30 *μ*m in diameter, was used (Multi Channel Systems, Reutlingen, Germany). The specific Multi Electrode Array (MEA) configuration was purchased according to our pre-defined design. In this array the nearest electrodes are spaced 990 *μ*m from each other. Recording of central region activity was performed using two columns of 4 electrodes separated by 800 *μ*m, located in the centre of the culture. The insulation layer (silicon nitride) was pre-treated with polyethyleneimine (Sigma, 0.01% in 0.1 M Borate buffer solution). A commercial amplifier (MEA2100, MCS, Reutlingen, Germany) was used, analog data was low-pass filtered (9 KHz) and pre-amplified with a ×11 gain. Analog to Digital converters with frequency limits of 100–5,000 Hz and a sampling rate of 50 KHz were applied. For a detailed description of the recording setup^60^. Data is transferred to a PC via USB2.0 connection using 16 bit format, in resolution of 27 nV and a rate of 25 KHz and analysed using Matlab (Mathworks, Natick, MA, USA).

### Analyses

Action potentials were detected on-line by threshold crossing (6 × standard deviation). The threshold value was defined separately for each of the recording channels at the beginning of an experiment, from a 2 sec long recorded voltage trace. A minimum of 6 ms interval between spikes was required, per electrode. Synchronous events were analysed off-line by threshold crossing of summed action potentials in 25 ms bins. Threshold value was adjusted relatively to 25% of active electrodes in the relevant recording area. The initiation time of activity in a given recording region was determined by the average time stamp of the first three spikes in that region. Synfire chains were detected as described by Schrader *et al*.^42^. Briefly, a pair-wise similarity matrix was computed by comparing the set of active electrodes in bins of 3 ms; values were scales between 0–1 by normalizing to the minimal number of active electrodes in each bin. The normalized matrix of similarity was then plotted, color coded such that black depicts full identity between two time bins, and a value of zero is depicted white. In the resulting image, a continuous 45 degree line is the signature of ongoing regularity, while the time gap between such lines represents the period of regularity.

## Additional Information

**How to cite this article**: Keren, H. and Marom, S. Long-range synchrony and emergence of neural reentry. *Sci. Rep.* **6**, 36837; doi: 10.1038/srep36837 (2016).

**Publisher's note**: Springer Nature remains neutral with regard to jurisdictional claims in published maps and institutional affiliations.

## Supplementary Material

Supplementary Information

Supplementary Information

Supplementary Information
