# Supplementary figures and images for: Long-range synchrony and emergence of neural reentry
Source: Sci Rep. 2016 Nov 22;6:36837. doi: 10.1038/srep36837 (PMC5118796; doi:10.1038/srep36837)

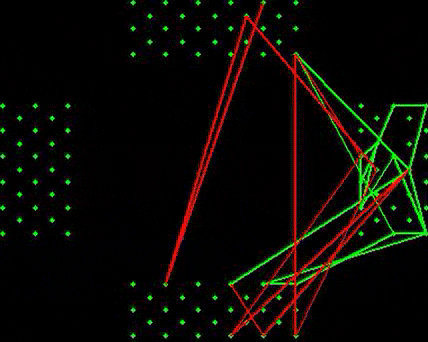

Supplement: Supplementary Information [file srep36837-s1.gif]

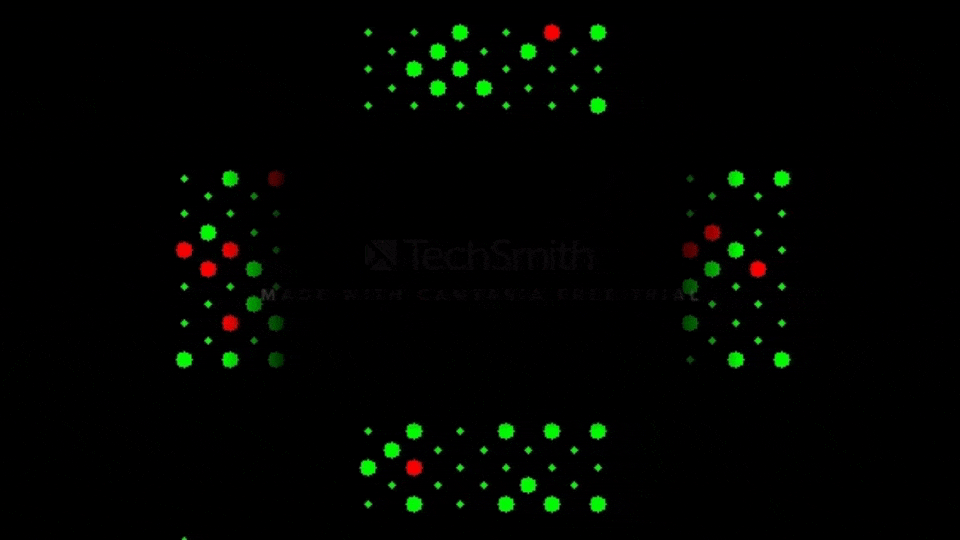

Supplement: Supplementary Information [file srep36837-s2.gif]
