# Supplementary material for: Long-range synchrony and emergence of neural reentry
Source: Sci Rep. 2016 Nov 22;6:36837. doi: 10.1038/srep36837 (PMC5118796; doi:10.1038/srep36837)
Supplement: Supplementary Information [file srep36837-s3.pdf]

Long-range synchrony and emergence of neural reentry

Hanna Keren[1,2,*] **and Shimon Marom**[1,2]

[1]Network Biology Research Laboratory, Electrical Engineering
[2]Department of Physiology, Biophysics and Systems Biology, Medicine
Technion - Israel Institute of Technology, Haifa 32000, Israel
[*]hannyk@tx.technion.ac.il

Supplementary videos

S1. Spatial long-range and reentry propagation. The spatial coordinates of activity position during propagation across a disinhibited network, before and during reentry. Each 10 consecutive spikes are presented simultaneously to demonstrate the spatial dispersion of activated connections (depicted red; the history of previous activations is shown in green).
S2. Spatial and temporal representation of convergence to reentry. The coordinates of activity position and scaled time delays between all spikes are presented (active electrodes are depicted red; previously active electrodes are shown in green). The propagation during five separate synchronous events is demonstrated, followed by the convergence to a slower propagation of cyclic reentry.
